# Immunogenicity to biological drugs in psoriasis and psoriatic arthritis

**DOI:** 10.6061/clinics/2021/e3015

**Published:** 2021-09-20

**Authors:** Fernando Valenzuela, Rodrigo Flores

**Affiliations:** I Department of Dermatology, School of Medicine, University of Chile, Santiago-Chile.; II Department of Dermatology, Clínica Las Condes, Santiago-Chile.

**Keywords:** Immunogenicity, Anti-Drug Antibodies, Anti-TNF, Anti-IL-12/23, Anti-IL-17, Anti-IL-23

## Abstract

Monoclonal antibodies or fusion proteins, defined as biological drugs, have modified the natural history of numerous immune-mediated disorders, allowing the development of therapies aimed at blocking the pathophysiological pathways of the disease, providing greater efficacy and safety than conventional treatment strategies. Virtually all therapeutic proteins elicit an immune response, producing anti-drug antibodies (ADAs) against hypervariable regions of immunoglobulins. Immunogenicity against biological drugs can alter their pharmacokinetic and pharmacodynamic properties, thereby reducing the efficacy of these drugs. In more severe cases, ADAs can neutralize the therapeutic effects of the drug or cause serious adverse effects, mainly hypersensitivity reactions. The prevalence of ADAs varies widely depending on the type of test used, occurrence of false-negative results, and non-specific binding to the drug, making it difficult to accurately assess their clinical impact. Concomitant use of immunosuppressors efficiently reduces the immunogenicity in a dose-dependent manner, either by decreasing the frequency of detectable ADAs or by delaying their appearance, thereby enhancing the effectiveness of biological therapies. Among the new therapeutic strategies for the management of psoriasis, biological agents have gained increasing importance in recent years as they interrupt key inflammation pathways involved in the physiopathology of the disease. Reports regarding ADA in new biologics are still scarce, but the most recent evidence tends to show little impact on the clinical response to the drug, even with prolonged treatment. It is therefore essential to standardize laboratory tests to determine the presence and titles of ADAs to establish their administration and management guidelines that allow the determination of the real clinical impact of these drugs.

## INTRODUCTION

In the last decade, several new treatment methods have been developed to attack various physiological mechanisms underlying inflammatory diseases. Monoclonal antibodies or fusion proteins, defined as biological drugs, have modified the natural history of numerous immune-mediated disorders, such as rheumatic disease, inflammatory bowel disease, systemic vasculitis, and psoriasis (1). These agents have allowed the development of therapies targeting pathophysiological pathways of diseases with even greater efficacy and safety compared to conventional treatment strategies (2). As they are exogenous molecules to the immune system, drug-associated immunogenicity could develop, leading to a significant impact both on the efficacy and safety of the treatment as well as the compliance and individualization of these therapies in certain patients. The immune response generated against monoclonal antibody therapies can result in low circulating drug levels, loss of therapeutic efficacy, poor drug survival, and/or associated adverse events, such as infusion reactions. Several factors can influence the clinical impact of this immunogenicity, and their identification can be useful for the optimization and personalization of biological therapies. Concomitant immunosuppressive therapy can significantly reduce the frequency of detection of anti-drug antibodies (ADAs) or delay their appearance (3). In this regard, it has been shown that the concomitant administration of methotrexate (MTX) or azathioprine (AZA) reduces the immunogenicity in a dependent manner, mainly with the use of tumor necrosis factor (TNF) inhibitors (4).

## WHAT ARE MONOCLONAL ANTIBODIES?

Monoclonal antibodies (mcABs) are proteins produced in vitro using recombinant techniques from a single clone of B lymphocytes. They were recognized for the first time in the sera from patients with multiple myeloma, in which the clonal expansion of malignant plasma cells generated high levels of a specific antibody subtype. The fusion of a murine B cell with an immortal myeloma cell generates a hybridoma that produces these antibodies. Murine mcAB has been genetically engineered to produce molecules with a higher proportion of human proteins. Currently, chimeric (65% human), humanized (> 90% human), and fully human (100% human) mcABs are available. The higher the percentage of murine proteins, the greater the ability of mcAB to induce an anti-mouse humoral immune response (*HAMA, human anti-mouse antibodies*) (1,5). mcAB is intended to mimic or inhibit the action of natural proteins, suppressing only a specific part of the immune system. They block interactions between the target molecules and their ligands, for example, by acting on specific mediators of inflammation or by triggering the lysis of the coated tumor cells. Many mcABs have been developed using recombinant DNA technology, and several are available on the market with a safety profile considered even more favorable than traditional immunosuppressive agents (5). Immunogenicity against biological drugs is manifested by the generation of ADAs that can alter their pharmacokinetic and pharmacodynamic properties, reducing the efficacy of the drug. In more severe cases, ADAs can neutralize the therapeutic effects of the drug or even cause serious adverse effects. Although some factors that contribute to the formation of ADAs are known, the molecular mechanisms by which therapeutic mcABs cause ADAs have not been completely clarified. Humanized mcABs unexpectedly show similar immunogenicity to chimeric antibodies, and based on their greater sequence homology, chimeric mcABs are sometimes more “human” than humanized mcABs, demonstrating the participation of other factors, different from the presence of murine genetic sequences, in the development of this immunogenicity (6).

## IMMUNOGENICITY TO BIOLOGICALS

Virtually all therapeutic proteins, known as biological drugs, elicit an immune response with the consequent production of ADAs. This phenomenon is the result of a specific adaptive immune response that involves the participation of T and B lymphocytes. Most of these antibodies are directed against the antigen-binding site of therapeutic mcABs and, therefore, neutralize AC. This ADA response explains why fully human antibodies can still be highly immunogenic (7). Two fundamental principles explain the theoretical basis for the immunogenicity of biologic agents: biopharmaceuticals are exogenous in nature (neo-antigens or non-self antigens) and may have little or no similarities with endogenous molecules, preventing the development of immune tolerance, so that the receptor immune system recognizes biological drugs as foreign molecules (8). It is now known that ADAs are predominantly directed against immunoglobulins’ hypervariable regions, known as complementarity determining regions (CDRs), which form the antigen-binding site of the therapeutic antibody. In this way, they elicit genuine neutralizing “anti-idiotypic” responses by competing with the drug's target molecule (e.g., TNF) for the drug binding site. The neutralization of ADAs directly affects the mechanism of action of the drug by preventing mcAB from binding to its target (9). Non-neutralizing ADAs, which bind to other parts of the drug, can also form immune complexes that can alter the clearance of the biological drug and/or reduce its bioavailability, lowering free drug concentrations. The presence of ADAs may be associated with two main clinical consequences: a reduction in therapeutic efficacy and/or an increased risk of adverse events (AE), mainly hypersensitivity reactions (9). The reason why ADAs are developed in different inflammatory diseases has not yet been elucidated; it could be related to the pathogenic mechanism of the disease itself or to the different degrees of cell activation (10,11).

Several studies have revealed the significant impact of immunogenicity on the response to biological drug treatment. Considering that the quantification of ADAs is a challenge, since there are different laboratory tests to evidence them, quantitative data are also difficult to compare among clinical studies. The prevalence of ADAs varies widely depending on the type of tests used as well as the frequency of false negative results and non-specific binding to the drug that can occur in some of them, making it difficult to accurately assess their clinical impact (12). Many factors can influence the immunogenicity findings, including sample handling, time of collection, concomitant medications, and underlying diseases (Table 1). In addition, methodological differences can substantially affect the results, without the complete understanding of the conditions that produce certain ADA titles. Thus, regardless of the incidence of ADAs, the actual antibody titers and their effects on the pharmacokinetics, efficacy, and safety are the most relevant points to consider (13).

Depending on the circulating ADA titers, a reduction in the drug concentration can be clinically significant. In patients with low titers of ADAs, drug concentrations may remain high enough to be effective, while in patients who develop high titers of ADAs, a substantial portion of the drug will be neutralized and is likely to produce a clinical non-response over time (21). The presence of ADA could reduce therapeutic responses by up to 80%, particularly in patients who do not receive concomitant MTX (22). MTX has been shown to be efficient in reducing the immunogenicity in a dose-dependent manner, either by reducing the frequency of detectable ADAs or by delaying their appearance, thereby increasing the effectiveness of biological therapies (4). Despite the fact that most ADAs do not cross-react with other biological agents with different CDR regions, patients generating ADAs to a biological drug will have a greater probability of developing ADAs to a new biological drug. In patients who do not respond to biological agents and who have developed ADAs, it is recommended to switch to a less immunogenic drug, regardless of the mechanism of action (23). The most common AEs associated with the presence of ADAs are hypersensitivity reactions, the severity of which can range from mild to severe. Although immunoglobulin (Ig)-E-type ADAs have been reported, the vast majority of ADAs belong to the IgG class, suggesting an alternative pathway (independent of IgE) of anaphylatoxin production that may or may not non-specifically activate the mast cells. These ADA-independent cytokine release syndromes can be managed in the short term by stopping the infusion of biological agents, decreasing the infusion rate, or administering histamine blockers and corticosteroids (24).

## BIOLOGICALS IN PSORIASIS AND PSORIATIC ARTHRITIS

Psoriasis (PsO) is a chronic, immune-mediated, inflammatory skin and systemic disease that affects approximately 2-3% of the world’s population (25). The different phenotypes of this entity are the result of genetic and epigenetic changes, ultimately determining an altered immune function and a dysregulated systemic inflammatory response (26). The chronic nature of the disease requires prolonged systemic therapy to maintain optimal clinical responses. In the last two decades, biological therapies have revolutionized the management of PsO and psoriatic arthritis (PsA), thanks to advances in the understanding of its pathogenesis. The initial PsO trigger is believed to involve the activation of antigen-presenting dermal dendritic cells and the production of interferons (IFN-α and IFN-β), the antimicrobial peptide LL-37 (cathelicidin), and TNF-α by damaged keratinocytes. In addition, the generation, maturation, and recruitment of various inflammatory cells, orchestrated by effector T helper lymphocytes (Th 17 and 22) through mediating cytokines, chemokines, and interleukins (IL), mainly represented by the TNF-α/IL-23/IL-17 axis (27,28). Among the new therapeutic strategies for the management of psoriatic disease (PD), biological agents have gained increasing importance in recent years, by interrupting key inflammation pathways in patients with PsO and PsA (29,30).

## CLINICAL EVIDENCE

With the advent of biologic drugs, the treatment of PD has changed dramatically owing to its high efficacy and tolerable safety. Currently, a variety of biological agents are available for the treatment of PsO and PsA in the long term; therefore, it is essential to understand the potential development of clinically relevant ADAs in the course of therapy (3). Although there are clear differences among varied therapeutic biologic products in terms of reported rates of ADAs, there is no guideline or consensus on an approach for managing these. Making valid comparisons of immunogenicity between different drugs is problematic, since there are different types of laboratory tests for the analysis of these ADAs. Furthermore, the patient population included, as well as the molecular structure of the biological drugs themselves, are highly influential in the prevalence of reported ADAs and the impact they generate on the clinical response (Table 2) (31).

### *Anti-TNF*:

Anti-TNF-α agents have demonstrated efficacy both in monotherapy and in combination with disease-modifying antirheumatic drugs (DMARDs) in the treatment of chronic immune-mediated inflammatory diseases, such as rheumatoid arthritis (RA), Crohn's disease (CD), PsO, and PsA (32). However, the immunogenicity of these drugs plays a significant role in the variability of clinical responses among patients with these types of diseases. The clinical impact on the outcome of anti-TNF-α drug treatments in PsO and PsA patients has not yet been completely clarified. Despite the high efficacy rates reported with these agents in PsO, a substantial proportion of patients still experience primary or secondary failure or develop significant side effects, potentially attributable to immunogenicity (33,34).

Infliximab (IFX) was the first anti-TNF-α approved by international regulatory agencies for use in patients with PD. It is an IgG1 chimeric mcAB that is administered intravenously. Meanwhile, adalimumab (ADL) and golimumab (GOL) are humanized mcAB, produced by recombinant DNA techniques and administered subcutaneously. Etanercept (ETN) is a fusion protein consisting of two extracellular receptor domains (TNFR2) and an Fc fragment of human IgG1. Certolizumab (CTL), on the other hand, is a humanized Fab fragment conjugated with polyethylene glycol (35). This peculiarity does not allow it to bind to the Fc receptor of fetal IgG, preventing its passage through the placental barrier or into breast milk, making its use safe in pregnant women (36).

The immunogenicity of these agents seems to be more related to the specific molecular structure of the anti-TNF-α agent and how it acts as a different immune stimulus. In this way, these ADAs affect the pharmacokinetics (PK) of anti-TNFs by binding to specific idiotypes of the drug, neutralizing their activity, and accelerating the clearance of the antibody-drug complexes by the reticuloendothelial system. Moreover, the existence of inflammatory mechanisms not mediated by TNF may also be responsible for the lack or loss of response to anti-TNF, as well as other factors that significantly affect the PK of anti-TNF, such as body surface, serum albumin concentration, degree of inflammation (TNF levels), and disease severity. The concomitant administration of antimetabolites, such as AZA or MTX, may increase the concentrations of anti-TNFs, reducing the formation of antibodies or the clearance of immune complexes (37). The clinical consequences of the development of ADAs are heterogeneous and include severe allergic/anaphylactic reactions and a reduction or loss of therapeutic efficacy (38).

Recently, Pecoraro et al. carried out a systematic review and meta-analysis that included 34 studies, enrolling 4273 patients affected by some autoimmune inflammatory disease under treatment with anti-TNF-α. In this group, the development of ADAs was evidenced in up to 18.6% of cases, with a marked reduction in clinical response (Response rate (RR) 0.43, 95% confidence interval (CI) 0.3-0.63), especially in patients treated with IFX (RR 0.37) or ADL (RR 0.40) (39). A retrospective cohort study from the ABIRISK project recruited a total of 366 patients with RA treated with ADL (n=240) or IFX (n=126). Of these, 92.4% were anti-TNF virgin (n=328/355) and 96.6% were treated with MTX (n=341/353). After a follow-up period of 18 months, ADAs were detected in 19.2% of patients treated with ADL and in 29.4% of patients in the IFX group. The cumulative incidence of ADAs increased over time to 50% and 66.7% for the ADL and IFX groups, respectively, at the end of the study period. The factors associated with a higher risk of developing ADA were a longer duration of disease, RA of moderate activity, and prolonged smoking habit (40).

PsO and PsA are other examples in which anti-TNFs, despite their high response rates, fail to demonstrate efficacy (primary failure) or induce significant side effects in a substantial proportion of patients. In placebo-controlled clinical trials, 40-60% of patients with active PsA treated with ADL or IFX and 30-40% of those who received ETN failed to meet the criteria of the American College of Rheumatology (ACR) for a clinical response improvement of at least 20% (ACR20) (33,41). Similarly, between 20% and 50% of patients with plaque PsO do not achieve clinical improvement of at least 75% of their baseline, evaluated using the Psoriasis Area and Severity Index (PASI). It has also been shown that only 75-85% of patients with PsO manage to maintain the long-term PASI75 response with anti-TNF agents used during the first period of treatment (34,42).

Regarding the immunogenicity of each drug in this particular group, it is worth mentioning.

#### *Infliximab (IFX)*:

In a study by Menter et al. in 2007, after 50 weeks of treatment with IFX, between 35-50% of psoriatic patients developed antibodies against the drug (43). The factors related to a greater development of ADAs were a dose of 3 mg/kg *versus* 5 mg/kg, intermittent drug administration regimens or as needed *versus* scheduled, and no association with MTX as concomitant therapy. On the other hand, the presence of antibodies against IFX was associated with infusion-related adverse reactions only in the retreated group of patients and after an interval of 20 weeks from the last administration (23% in patients positive for ADAs, compared to 8% in patients without ADAs). Furthermore, patients with antibodies were less likely to maintain a response at week 50 of follow-up (43-45). Similarly, in patients with active PsA treated with IFX, doses of 5 mg/kg were related to ADA production in up to 15.4% of cases, after 54 weeks with the drug. The development of anti-IFX antibodies was more frequent in patients who did not receive associated treatment with MTX at the beginning of the study (26.1% *versus* 3.6% in the patients who did receive it), showing an inverse correlation with the clinical response. The median percentage improvement in ACR20 for ADA-positive patients was lower (21.7%) than in those who did not develop antibodies (33.3%) at the end of the study (46).

#### *Adalimumab (ADL)*:

Regarding ADL, the reports are more limited, although several studies mention an incidence of ADAs between 6% and 45%, depending on the technique used for their detection, these would not be neutralizing. The presence of anti-ADL antibodies was linked to a decrease in the efficacy of the drug to achieve PASI75 (23.1% *versus* 72.7% in ADA-negative patients), with rapid loss of response (PASI <50) at week 52 of follow-up (47). Vogelzang et al. observed that ADL concentrations were significantly lower at 28 and 52 weeks of follow-up in 103 patients with PsA and positive ADAs, correlating in the same way with a lower clinical response. ADL concentrations reflect the amount of drug available in the serum that binds to its target molecule. If no free drug concentration is available or insufficient, inflammation cannot be effectively suppressed. Therefore, measuring drug concentrations in patients who do not respond adequately could provide more information on why there is an inadequate response (48).

#### *Etanercept (ETN)*:

Etanercept is believed to be less immunogenic than other anti-TNF agents (42). In patients with PsO, the frequency of detection of anti-ETN antibodies varies between 1.5% and 2.8%, although in open label extension studies of up to 96 weeks of follow-up, they were evidenced in up to 18.3% of the patients. Consistent with the results of previous clinical trials, these ADAs were shown to be non-neutralizing and to have no apparent effect on the efficacy of the drug or its safety profiles (49).

#### *Golimumab (GOL)*:

Studies with GOL in the treatment of patients with RA, PsA, and ankylosing spondylitis report low ADA titers, without impact on clinical efficacy or adverse reactions at the injection site (50).

#### *Certolizumab (CTL)*:

CTL pegol is a useful and safe option for the treatment of moderate to severe severity plaque PsO, and it provides an important treatment option for women of childbearing age, where the available options are limited (51,52). The reported incidence of ADA for CTL varies in the different studies between 5-37% depending, in large part, on the method used for its identification, with mixed results regarding the clinical response (no effect in patients with RA, but with reduced effectiveness in CD) (53). Although the proportion of patients with detectable anti-CTL antibodies may be high, the drug concentration is above the therapeutic range (> 20 mg/L), which is correlated with the ability to neutralize TNF (54). In phase III studies carried out in patients with plaque PsO treated with 200 mg or 400 mg of CTL, the presence of ADAs was demonstrated in 19.2% and 8.3%, respectively, on one or more occasions at the 48th week of follow-up. However, the presence of these factors did not appear to be associated with an increase in AE (52,55,56).

### Anti-IL-12 and 23:

IL-12 and 23 participate in PsO pathogenesis by facilitating the inflammatory Th1 response. IL-12 is a heterodimeric cytokine composed of two subunits, p50 and p40. This latter subunit is also part of the IL-23 receptor, which is a common component of both ILs (57). Ustekinumab (UTK) and Briakinumab (BAK), two types of mcAB directed against the p40 subunit of IL-12/23 were developed and evaluated as therapeutic alternatives for PsO and other immune-mediated diseases. UTK is the only IL-12/23p40 inhibitor approved by the Food and Drug Administration (FDA) for the treatment of moderate to severe plaque PsO and PsA. The clinical development of BAK was discontinued due to safety concerns reported in clinical trials, including cardiac events and malignancies (58).

#### *Ustekizumba (UTK)*:

It is an IgG1/k type mcAB, humanized, with high affinity, directed against the p40 subunit of IL-12/IL-23. It mainly inhibits the Th17 lymphocyte signaling pathways, approved by the FDA for the treatment of moderate to severe PsO since September 2009 and PsA since September 2013 (59). Recently, Hanauer et al., in a long-term follow-up study (5 years) in patients with CD treated with subcutaneous UTK, demonstrated a low incidence of ADA formation (4.6%) at week 156, with maintenance of the clinical response and good tolerance (60). On the other hand, Leonardi et al. in a phase III, randomized, double-blind study, PHOENIX 1, of UTK controlled by placebo, in which 766 patients with moderate to severe PsO were recruited, showed that 38 of the 746 patients who completed the protocol and remained on the drug (5.1%) developed ADAs at low titers (<1/320) at week 76, which were not related to adverse reactions at the injection site (61,62).

### Anti-IL 17:

IL-17 plays a fundamental role in the pathogenesis of PsO and PsA, and is part of a family of cytokines that includes six members (IL-17A, IL-17B, IL-17C, IL-17D, IL-17E, and IL-17F). IL-17A is considered the most important, since by interacting with its receptor (IL-17R), it produces chemoattraction of neutrophils, recruitment of T helper-17 lymphocytes and stimulation of macrophages, endothelial cells, and fibroblasts, perpetuating the inflammatory response (63). To date, three antagonists of the IL-17 pathway have been approved by the FDA for the treatment of PsO and PsA: secukinumab (SCK), ixekizumab (IXK), and brodalumab (BDL). They were supported by phase III clinical studies, demonstrating high efficacy, tolerability, and safety (29).

#### *Secukinumab (SCK)*:

It is a fully human anti-IL-17A mAb that has demonstrated efficacy in the treatment of PsO in moderate to severe plaques. In seven double-blinded, randomized (DBR) phase III studies, statistically significant superiority of SCK was demonstrated from week 12 of treatment, when compared with placebo in clinical responses to PASI75/90/100, *Investigator Global Assessment* (IGA) 0/1, ACR20/50, and quality-of-life indices, such as the *Dermatology Life Quality Index* (DLQI). It is an effective and safe drug, with rapid and long-lasting clinical responses, across the spectrum of manifestations of PsO. Although the incidence rate of AE is low and comparable with that of other biological agents, a higher incidence of mucocutaneous infections by yeast of the *Candida* genus stands out. This is probably explained by the fact that IL-17A plays a key role in mucocutaneous microbial surveillance, stimulation of granulopoiesis, and neutrophil trafficking. SCK has shown low immunogenicity in vitro and in clinical trials. In phase III clinical studies, only 0.4% of patients (10/2842) developed ADAs, the majority non-neutralizing, without evidence of modification in the pharmacokinetics, safety, and efficacy of the drug, although the small number of patients limited the power of the study (64). Recently, Reich et al. evaluated the immunogenicity of SCK in a 5-year follow-up period. Of a total of 1821 patients, 1636 were subjected to analysis for emerging ADAs with treatment, of which only 32 patients developed anti-SCK antibodies, which determined an incidence of less than 1% of new ADAs per year. Neutralizing antibodies were detected in 9 of the 32 patients, half of whom were transient in duration. As an important conclusion, the researchers emphasized that no titer or type of antibodies affected the efficacy, safety, or pharmacokinetics of SCK (65).

#### *Ixekizumab (IXK)*:

IXK is a humanized IgG4/k type mcAB, with high selectivity against IL-17A, approved since 2016 by the FDA and the European Medicines Agency (EMA) for the treatment of moderate to severe plaque PsO, and in 2017, the FDA also approved its indication in PsA (29). The therapeutic efficacy of IXK was demonstrated in DBR clinical trials, offering rapid and sustained disease control, achieving PASI75 and PASI90 response rates of approximately 90% and 70% of patients, respectively, at 12 weeks of treatment. In the long term, approximately 80.5% of patients maintained PASI75 after 3 years of follow-up. In head-to-head studies against ETN, UST, and GSK, IXK's superiority in achieving PASI100 was also demonstrated in up to 40% of cases at week 12. Similar response rates were observed in patients with initial scalp, nail, or palmoplantar involvement, with a good safety profile and prolonged use (66). Regarding anti-IXK antibodies, Blauvelt et al. in 2016 evaluated in a blind, randomized and controlled way, the presence of ADAs in patients treated with IXK during the induction (weeks 0-12) and maintenance (weeks 12-60) period. Treatment-induced serum ADA levels were divided into subgroups according to antibody titers (negative, low, moderate, and high). At 12 weeks, the vast majority of patients were negative for ADAs, 91.0% in those who received IXK every 2 weeks, and 86.6% in those with IXK every 4 weeks. Patients who developed anti-IXK antibodies at 12 weeks had low titers of 5.7% and 8.0%, moderate titers between 1.6% and 3.0%, and high titers in 1.7% and 2.4% of cases, depending on whether they received the drug every 2 or 4 weeks, respectively. When evaluating clinical efficacy during the induction period in patients who received IXK every 2 weeks, only those with high titers of ADAs had reduced responses in PASI75, compared to negative patients for ADAs, with an average drop of 53.5% of clinical response for IXK patients every 4 weeks, compared to 36.8% for IXE every 2 weeks. The important thing about this study was that at the end of the 60 weeks of follow-up, the clinical efficacy of IXK was similar among all groups of patients with ADA, regardless of the administration interval and without being associated with other AE (67). Recently, these results were corroborated by Reich et al. in a study with similar characteristics (68).

#### *Brodalumab (BDL)*:

This recombinant human IgG2 mcAB of subcutaneous injection has a high affinity for the IL-17 receptor A. Unlike other anti-IL-17 molecules, it not only acts by blocking the biological activity of the pro-inflammatory cytokines of the IL-17 family (IL-17 A, IL-17C, and IL-17F), but also anti-inflammatory cytokines, such as IL-17E. It was approved by the FDA in February 2017 to treat patients with moderate to severe PsO. In Phase III clinical trials, AMAGINE-2 and AMAGINE-3, conducted in 2015, Lebwohl et al. compared BDL with UST *versus* placebo. From the start of the study to week 52, 28 (1.8%) AMAGINE-2 protocol patients and 37 (2.3%) AMAGINE-3 patients developed anti-BDL antibodies during the course of treatment (69). In a recent publication, Bagel et al. analyzed data from phase I, II and III studies on the use of BDL in PsO, with the objective of evaluating the potential effects of anti-BDL antibodies regarding safety, efficacy and percentage of retreatment during an observation period at 12 and 52 weeks. Of the 4461 cases analyzed, ADAs were detected in only 2.7% of the patients. These had a transient persistence in 1.4% of the cases, and there was no development of neutralizing ADAs. Among ADA-positive patients, 60% achieved a score of 0 or 1 on the Static Physician's Global Assessment (sPGA) scale at week 12, in the group that received BDL 210 mg every 2 weeks, compared to 79.1% of patients who did not develop ADAs. All patients who experienced disease relapse, defined as sPGA> 3, were treated again with BDL 210 mg every 2 weeks (none of them positive for ADA), achieving an improvement of at least 75% in their PASI baseline. Although it is true, the authors emphasize that it is difficult to draw definitive conclusions regarding the effect of ADAs on the clinical response rate, and given the small number of patients with positive anti-BDL antibodies, the presence of these antibodies does not seem to be associated with the development of tolerance to the drug, as shown by the high percentage of patients with ADA that maintains efficacy at 52 weeks (70).

### Anti-IL-23:

IL-23 is secreted by tissue-resident dendritic cells and macrophages and is a key cytokine involved in the protective immune response against fungal and bacterial infections. However, a decrease in its production, observed in PsO, activates the inflammatory cascade early, maintains the phenotype of Th17 lymphocytes, and is critical in the production of pro-inflammatory cytokines, such as IL-17A, IL-17F, and TNF. To date, four mcABs have been developed that selectively and highly specifically block the action of IL-23 (71-73).

#### *Guselkumab (GSK)*:

GSK is a fully human IgG1/l mcAB that is directed against the p19 subunit of IL-23. It has been approved in Japan, since 2016, for the treatment of PsO vulgaris, PsA, pustular PsO, and erythrodermic psoriasis and by the FDA for the treatment of moderate to severe plaque PsO, since July 2017 (59). Two phase III studies comparing GSK with ADL in the treatment of moderate to severe PsO demonstrated the superiority of GSK in achieving improvements in PASI90 and IGA 0/1 at week 28 of follow-up, with persistence of the response in sustained therapy *versus* withdrawal of the drug, from weeks 28-48. Regarding anti-GSK antibodies, the VOYAGE 1 study reported the presence of ADAs in 5.3% of patients (26/492) at week 44, with generally low titers (81% with < 1:320), which were not associated with a reduction in the clinical efficacy of the drug or with reactions at the injection site (74). Similarly, in the VOYAGE 2 study, anti-GSK antibodies were detected in 57 of 869 patients (6.6%) at week 48, to generally low titers (88% with <1/160), which also did not affect the clinical response to treatment or the incidence of adverse reactions to injection (75). In a recently published letter to the editor, Zhu et al. presented the results from VOYAGE 1 and 2, a 100-week follow-up study of the same patients. Of the 1713 patients exposed to GSK, 8.5% developed ADAs transiently, with 76% of the cases having low (< 1:160), 11.6% medium (1:320), and 12.3% high titers (> 1:640), and only in 9 of 146 patients (6.2% of cases) were neutralizing antibodies. Regardless of the nature of the anti-GSK antibodies, no loss of clinical response could be demonstrated; therefore, no drug dose adjustments were necessary (76).

#### *Tildrakizumab (TDK)*:

TDK is a humanized IgG1/k mcAB with high affinity against the p19 subunit of IL-23, which can be administered intravenously or subcutaneously (59). At doses of 100 and 200 mg administered in weeks 0 and 4 and then every 12 weeks, it has demonstrated efficacy and safety in the treatment of chronic plaque PsO of moderate to severe severity. Recently, TDK has been approved for use in the treatment of chronic plaque PsO by the FDA and EMA (77). In a prospective study with pooled data from phase III clinical trials (P05495, reSURFACE 1 and reSUR-FACE 2), in patients with chronic plaque PsO, treated with TDK, Kimball et al. evaluated both the development of treatment-emergent ADA, as well as neutralizing antibodies and the effects that these could have on the pharmacokinetics, efficacy and safety of the drug. In this integrated analysis, emerging ADAs were observed in approximately 4% of the 1,400 evaluable patients who received TDK for 12-16 weeks and in approximately 7% of the 780 patients who used the drug continuously for 52-64 weeks. Similarly, the incidence of neutralizing antibodies was 2-5% with 100 mg and 2-3% with 200 mg of the drug for the same periods analyzed. This subgroup experienced a moderate decrease in TDK pharmacokinetics, with a reduction in clinical response determined by a significant 10-15% drop in the mean PASI score relative to patients without ADAs at 52 weeks. The development of ADAs was not associated with an increase in severe AEs or discontinuation of treatment. Overall, the incidence of potential immunogenicity-related AEs did not show a clear trend in patients with inconclusive titers or in any category of ADA-positive patients, compared to patients without ADA, similar to other results with anti-IL-23/IL-17 biologics (78).

#### *Risankizumab (RSK)*:

This biological agent is a humanized IgG1 mcAB that selectively binds to the p19 subunit of heterodimeric IL-23. In 2019, RSK received approval in Japan for use in treating adults with PsO vulgaris, PsA, generalized pustular PsO, and erythrodermic PsO, and in Canada, the United States, and Europe for patients with moderate to severe PsO. Among patients treated with RSK 150 mg for up to 52 weeks (n=1079), the presence of ADAs and neutralizing antibodies was detected in 24% (263) and 14% (150), respectively. In most cases, ADAs were not associated with changes in the clinical response or safety. High ADA titers in approximately 1% of RSK-treated patients were associated with a slight reduction in the clinical response. The incidence of drug injection site reactions was 3% in patients with ADAs *versus* 1% in those without ADA development at weeks 16 and 5 *versus* 3% from week 52 onwards (79).

## CONCLUSIONS

Currently, a variety of biological agents are available for the long-term treatment of PsO and PsA. These drugs, mcAB or fusion proteins, have been developed rapidly in recent decades, managing to revolutionize the treatment of inflammatory pathologies with high systemic repercussions, with the ultimate goal of interfering with key pathways in the inflammation cascade with high efficacy and safety, thanks to a more complete understanding of the pathophysiology underlying these diseases. Being exogenous molecules, they are capable of activating the immune system and triggering a specific adaptive immune response, producing specific neutralizing antibodies directed against the antigen-binding site of therapeutic mcABs. Factors derived from the drug itself as well as the patient and even the treatment regimen, influence the development of this immunogenicity. It is currently considered that the presence of ADA could reduce therapeutic responses by up to 80%, particularly in patients who do not receive immunosuppressive drugs concomitantly. Of the biologics indicated for the treatment of PD, anti-TNF drugs, particularly IFX, have the highest rates of ADAs, associated with loss of clinical effectiveness and higher incidence of adverse reactions to drug infusion, in low dose treatments, intermittent administration, or non-association with MTX, among others. Reports regarding ADA in new biologics are still scarce, but the most recent evidence suggests little impact on the clinical response to the drug, even with prolonged treatment. It is therefore essential to standardize laboratory tests to determine the presence and titles of ADAs to establish their administration and management guidelines that allow the determination of the real clinical impact of these drugs.

## AUTHOR CONTRIBUTIONS

Valenzuela F was responsible for the research design and conception. Flores R was responsible for manuscript writing. Valenzuela F and Flores R were responsible for the critical revision of the manuscript for important intellectual content.

## Figures and Tables

**Table 1:** Factors that influcence the immunogenicity of biologic agents.

Patient-related:	Genetic factors	*IL-10* gene polymorphism (fundamental in AB synthesis) in patients with rheumatoid arthritis (RA) treated with the anti-tumor necrosis factor (anti-TNF). Specific human leukocyte antigen (HLA) haplotypes: - HLA DRB1 in antigen-presenting dendritic cells in patients with hidradenitis suppurativa (HS) and adalimumab (ADL), inflammatory bowel disease (IBD), and infliximab (IFX). - HLA DBbeta-11, HLA-DQ-03, and HLA DQ-05 anti-NF alleles. V158F functional polymorphism in one of the FcgammaR genes that affects the AB binding capacity to the drug (eg. IFX in EC) (1).
	Disease type and activity	Immune system (IS) activation: - Due to immunoreactivity of the disease itself. - High expression of costimulatory molecules in dendritic cells that accelerate the production of anti-drug antibodies (ADAs). - B lymphomas in patients with RA treated with rituximab (RTX): ADA in 1-4% Type of inflammatory diseases: - Primary Sjörgen syndrome + *ANCA* (+) vasculitis: ADA in 25% of the patients - LES: ADA up to 40% - ADA anti-IFX in patients with RA (+) *versus* RA (-) = 62.5% *versus* 37.5%, respectively Reduced disease activity allows higher levels of circulating monoclonal antibodies (mcABs), which may promote immune tolerance
Drug-related:	Drug dose (and plasma concentration)	Lower doses>higher doses: Higher doses of the drug reduce immunogenicity and induce tolerance by depletion of the immune response (14)
	Route of administration	Intradermal or SBC>intravenous: Favor the uptake and presentation by antigen-presenting cells (APCs)
	Frequency of administration	Intermittent treatment>continuous therapy: Continuous administration allows the development of immune tolerance
	Chemical formulation	Molecular structure not identical to endogenous immunoglobulin (Ig) even in fully human mcAB (new epitopes in complementarity determining region (CDR) sequences) due to idiotype/anti-idiotype interactions (1). Severe anaphylactic reactions to cetuximab due to non-human carbohydrate residues that cross- react in red meat proteins sensitized patients (beef, pork, or lamb) that induce the formation of IgE-type ADA (15).
	Post-translational modifications	Removal of N-terminal glycosylation of the Fc fragments chains decreases the immunogenicity of mcAB. Impurities in the formulation processes. Danger model: IS responds more to substances that cause harm than exogenous ones. For example, impurities or residues in the processing of biologicals agents (1).
	Target molecules	Anti-IL-6 mcAB, tocilizumab, has low incidence of ADA formation because interleukin (IL)-6 participates in modulating the humoral immune response. mcAB directed at certain target molecules on cell surfaces, would induce greater immunogenicity than those directed against soluble molecules, since the latter require more processing to be finally presented as antigens Rituximab, chimeric anti-CD20 mcAB, selectively depletes CD20 (+) B lymphocytes, but does not affect pre-B or immature B cells lymphocytes, nor does it affect the maturation of memory plasma cells, which prevents the production of ADAs (16).
Treatment-related:	Treatment duration	Short>long treatments: - ADA titers decrease over time in prolonged treatments by inducing immunological tolerance due to continuous exposure to the drug.
	Treatment interruption	Variable response: - Greater development of ADA after temporary suspension of IFX *versus* continuous administration, without interruptions (39% *versus* 16%), in patients with Crohn's disease (CD) and ulcerative colitis (UC) (17). - Repeated cycles with several interruptions of omalizumab in patients with UC showed complete remission without development of ADA (18).
	Concomitant use of immunosuppressants	Methotrexate (MTX) favors the elimination of antigen-activated lymphocytes and/or stimulates the activity of regulatory T cells lymphocytes, avoiding clonal expansion of B cells. Dose-dependent effect on RA (19). The addition of azathioprine (AZA) in patients with inflammatory bowel disease (IBD) and loss of response to a first anti-TNF, favors the pharmacokinetic profile of a second anti-TNF (20).

**Abbreviations:** SI: Immune system; ANCA: Anti-neutrophilic cytoplasmic autoantibodies; IL: Interleukins; ADA: Anti-drug antibodies; mcAB: Monoclonal antibody; CDR: Complementarity determining region; MTX: Methotrexate; AZA: azathioprine; SLE: Systemic lupus erythematosus; RA: Rheumatoid arthritis; HS: Hidradenitis suppurativa; IBD: Inflammatory bowel disease; CD: Crohn's disease; UC: Ulcerative colitis; TNF: Tumor necrosis factor; ADL: Adalimumab; IFX: Infliximab; RTX: Rituximab; SBC: Subcutaneous; IV: intravenous; Ig: Immunoglobuilin.

**Table 2:** Monoclonal antibodies approved for the treatment of psoriasis and psoriatic arthritis, and the anti-drug antibodies (ADAs) rates reported for them (1,34).

mcAB	Target Molecule	Format	Indication	%ADA	%ADA neut
*ADL*	TNF-α	IgG1 human	RA, PsO	28%, 6-45%	no report
*BDL*	IL-17R	IgG2 human	Plaque PsO	2.7%	0%
*GLM*	TNF-α	IgG1 human	RA and PsA	31.7%	no report
*GSK*	IL-23 p19	IgG1 human	PsO placa	5.5%	0.4%
*IFX*	TNF	IgG1 chimeric	CD	66.7% cumulative in RA 5.4-43.6% PsO	no report
*IXK*	IL-17a	IgG4 humanized	PsO	9%	no report
*RSK*	IL-23 p19	IgG1 humanized	Plaque PsO	24%	14%
*SCK*	IL-17a	IgG1 human	PsO	0.41%	0.2%
*TDK*	IL-23 p19	IgG1 humanized	Plaque PsO	4.1-8.8%	0.6-3.34%
*UTK*	IL-12/23	IgG1 human	PsO	6.5%	no report

**Abbreviations:** ADA neut: neutralizing anti-drugs Antibodies; mcAB: Monoclonal antibody; ADL: Adalimumab; BDL: Brodalumab; GLM: Golimumab; GSK: Guselkumab; IFX: Infliximab; IXK: Ixekizumab; RSK: Risankizumab; SCK: Secukinumab; TDK: Tildrankizumab; UTK: Ustekizumab.
